# Construction of an oxidative phosphorylation-related gene signature for predicting prognosis and identifying immune infiltration in osteosarcoma

**DOI:** 10.18632/aging.205650

**Published:** 2024-03-20

**Authors:** Peng Zhou, Jin Zhang, Jinyan Feng, Guowen Wang

**Affiliations:** 1 Department of Bone and Soft Tissue Tumors, Tianjin Medical University Cancer Institute and Hospital, National Clinical Research Center for Cancer, Key Laboratory of Cancer Prevention and Therapy, Tianjin’s Clinical Research Center for Cancer, Tianjin, China; 2 Department of Orthopedics, Affiliated Hospital of Chifeng University, Chifeng, Inner Mongolia, China

**Keywords:** oxidative phosphorylation, osteosarcoma, prognosis, immunity, gene

## Abstract

Background: Osteosarcoma is a prevalent malignant tumor that originates from mesenchymal tissue. It typically affects children and adolescents. Although it is known that the growth of osteosarcoma relies on oxidative phosphorylation for energy production, limited attention has been paid to exploring the potential of oxidative phosphorylation-related genes in predicting the prognosis of individuals suffering from osteosarcoma.

Methods: All the data were retrieved from the UCSC Xena and GEO (GENE EXPRESSION OMNIBUS). Identification of the oxidative phosphorylation genes linked to the prognosis of individuals with osteosarcoma was done by means of univariate COX and LASSO regression analyses. Following that, patients were categorized into a high-risk group and a low-risk group as per the risk score determined by the identified oxidative phosphorylation genes. Furthermore, a comparison was made in terms of the survival and immune infiltration between both groups, and the prognostic model was established.

Results: Five oxidative phosphorylation genes (ATP6V0D1, LHPP, COX6A2, MTHFD2, NDUFB9) associated with the prognosis of individuals with osteosarcoma were identified and the risk prognostic models were constructed. In the current research, the analysis of the ROC curves indicated a superior predictive accuracy exhibited by the risk model. The prognosis was adversely affected by immune infiltration in the high-risk group in comparison with the low-risk group. The function of the oxidative phosphorylation-related prognostic gene set was verified by GO and KEGG analysis. Furthermore, the link between oxidative phosphorylation-related genes and osteosarcoma immune infiltration was examined by GSEA analysis.

Conclusions: In this study, a prognostic model that demonstrated good predictive performance was constructed. Additionally, this study highlighted a correlation between oxidative phosphorylation-related genes and immune infiltration.

## INTRODUCTION

The most frequent type of primary bone cancer in young individuals is osteosarcoma. The annual incidence of osteosarcoma is about 3-4 cases per million people [1]. Although the incidence of osteosarcoma is low, the degree of malignancy is high and the age of onset is low. Currently, the primary treatment methods for osteosarcoma include surgery, chemotherapy (neoadjuvant chemotherapy, adjuvant chemotherapy), radiotherapy, immunotherapy, and targeted therapy [2, 3]. Patients diagnosed with primary osteosarcoma in the absence of metastasis have a 5-year survival rate ranging from 65-70% [4]. In contrast, patients who experience metastasis or recurrence have an overall survival (OS) rate of just 20-30% [2, 3]. Therefore, finding the prognostic genes of osteosarcoma and establishing a prognostic model have important guiding significance for clinical treatment.

Changes in metabolic patterns are a crucial characteristic of cancerous cells. In 1924, Otto Warburg, a German biochemist, was the first to propose that tumor cells often utilize glycolysis for energy even under sufficient oxygen levels. This phenomenon is commonly referred to as the Warburg effect or aerobic oxidative phosphorylation [5, 6]. It is generally accepted that tumors obtain energy primarily through glycolysis. In fact, cellular metabolic pathways mainly include glycolysis, fat metabolism, glutamine decomposition, and oxidative phosphorylation. However, recent research has highlighted the essential role of oxidative phosphorylation in tumorigenesis and progression [7–10].

The current research aimed at identifying the oxidative phosphorylation genes linked to the prognosis of individuals with osteosarcoma. Additionally, it also aimed at establishing and verifying a prognostic model for the prediction of the overall survival rate of patients. Furthermore, the association of oxidative phosphorylation-related genes with the immune status in osteosarcoma was explored. The goal was to provide valuable insights into clinical treatment strategies.

## RESULTS

### Training and validation data sets of osteosarcoma patients

The flowchart of this article is shown in Supplementary Figure 14. The current investigation merged the clinical and transcriptional data of 88 individuals with osteosarcoma retrieved from the UCSC Xena database. A total of 84 individuals diagnosed with osteosarcoma, along with their clinical information, were included, after excluding samples with unreported or zero survival time. The dataset of GSE21257 contained 53 cases, and the gene expression matrix obtained from the combination of clinical data and transcriptional data was used as the validation set. The clinical data of the two groups are shown in Table 1.

**Table 1:** Summary of clinical data from the TARGET and GSE21257 osteosarcoma datasets.

	**Training set (TARGET n = 84)**	**Validation set (GSE21257 n = 53)**
Age (%)		
<18	66(78.6%)	34(64.2%)
≥18	18(21.4%)	19(35.8%)
Gender (%)		
Female	37(44.0%)	19(35.8%)
Male	47(56.0%)	34(64.2%)
Metastasis (%)		
No	63(75.0%)	19(35.8%)
Yeah	21(35.0%)	34(64.2%)
Survival (%)		
Alive	57(67.9%)	30(62.3%)
Dead	27(32.1%)	23(37.7%)

### Prognosis-related oxidative phosphorylation genes and risk-scoring model of osteosarcoma

**Figure 1:**
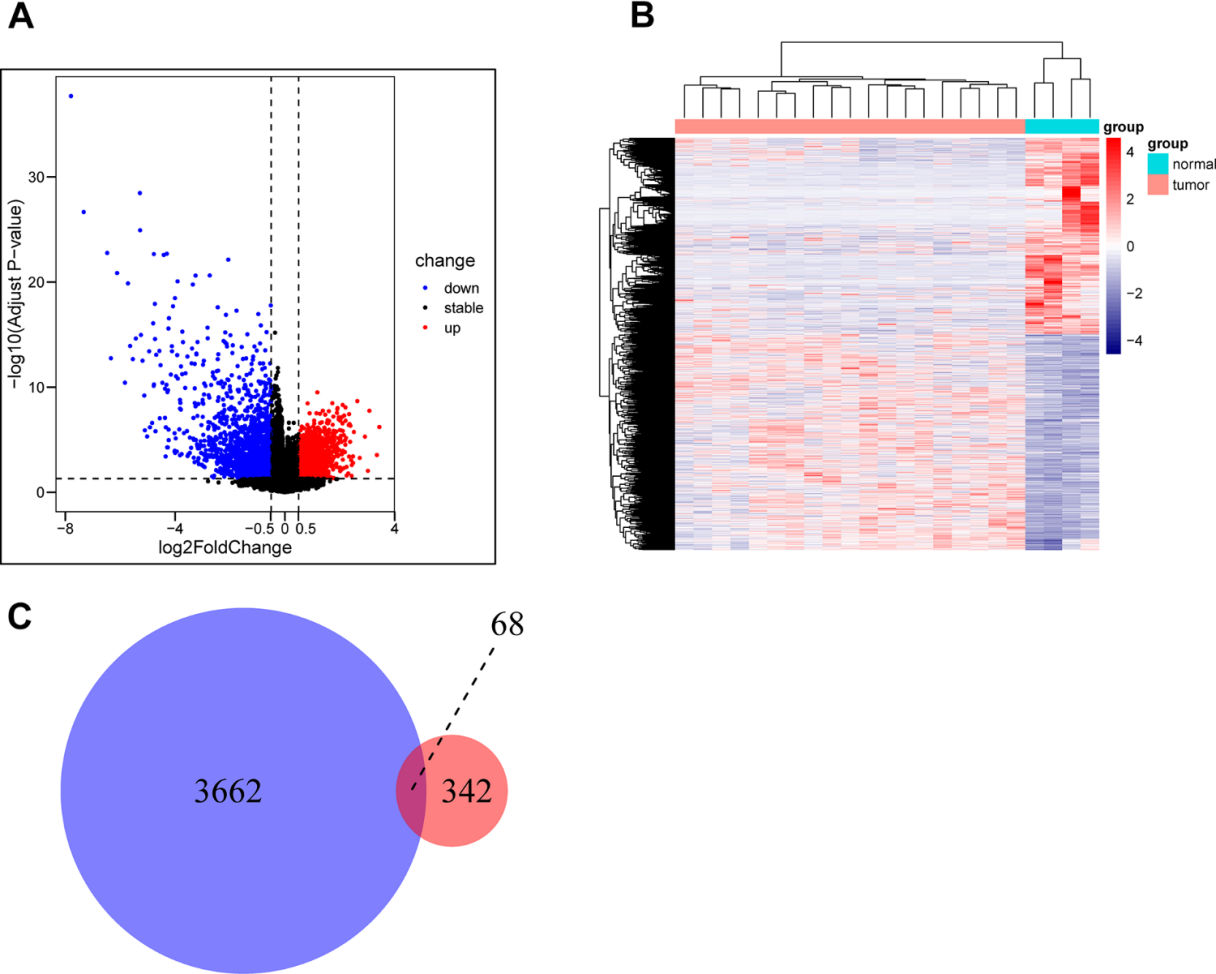
**Acquisition of oxidative phosphorylation-related genes.** (**A**, **B**) GSE28425 data set: Analysis of differences between osteosarcoma and adjacent cancer (osteoblasts) (differential gene volcano map and differential gene heat map). (**C**) Obtaining a Wayne map of differentially expressed genes related to oxidative phosphorylation in osteosarcoma.

**Figure 2:**
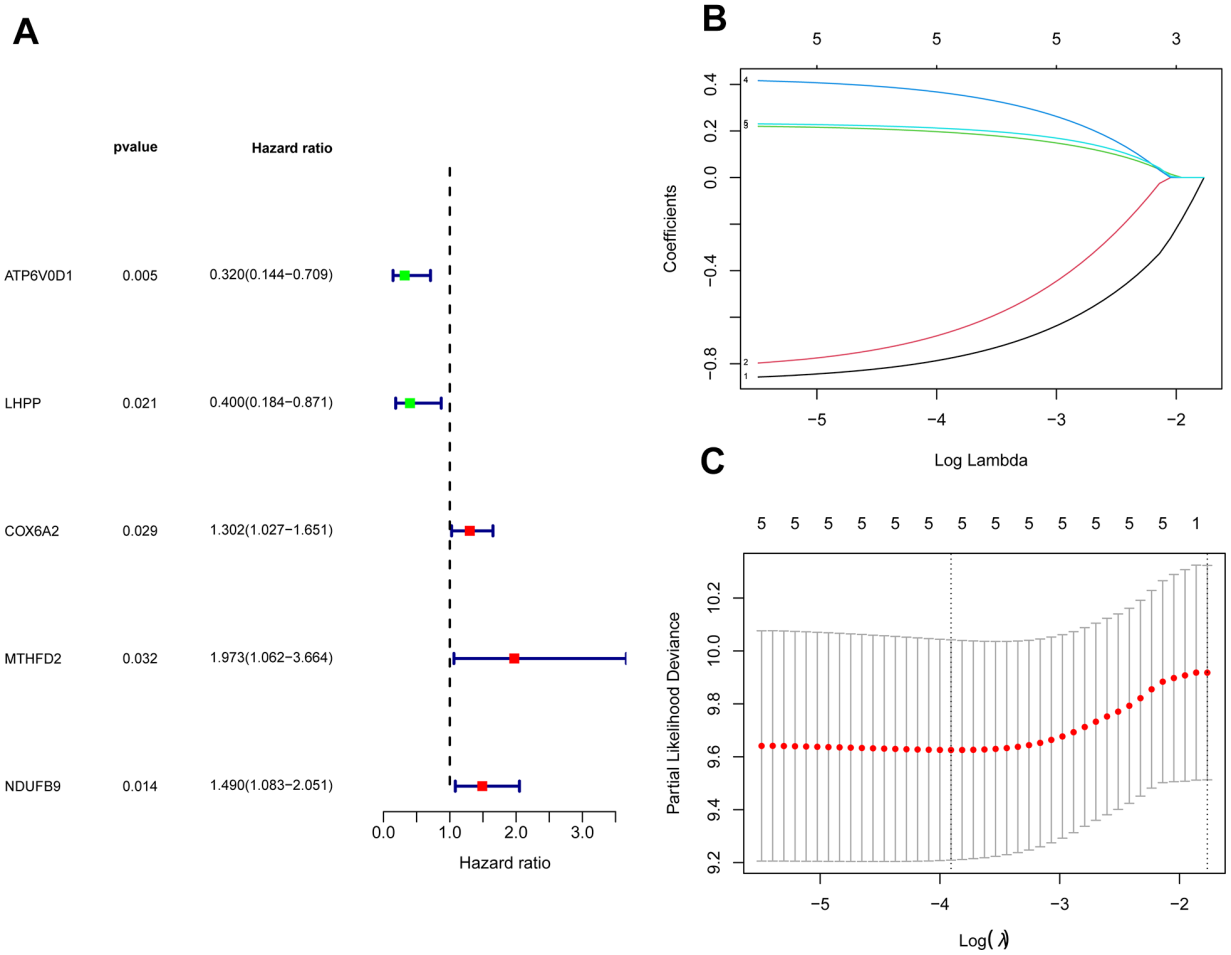
**Construction of a risk-scoring model for patients with osteosarcoma based on oxidative phosphorylation genes.** (**A**) Forest plots based on univariate Cox regression analysis showed that there were 5 oxidative phosphorylation genes with prognostic significance (*p* < 0.01). (**B**, **C**) LASSO regression to construct a risk model.

**Figure 3:**
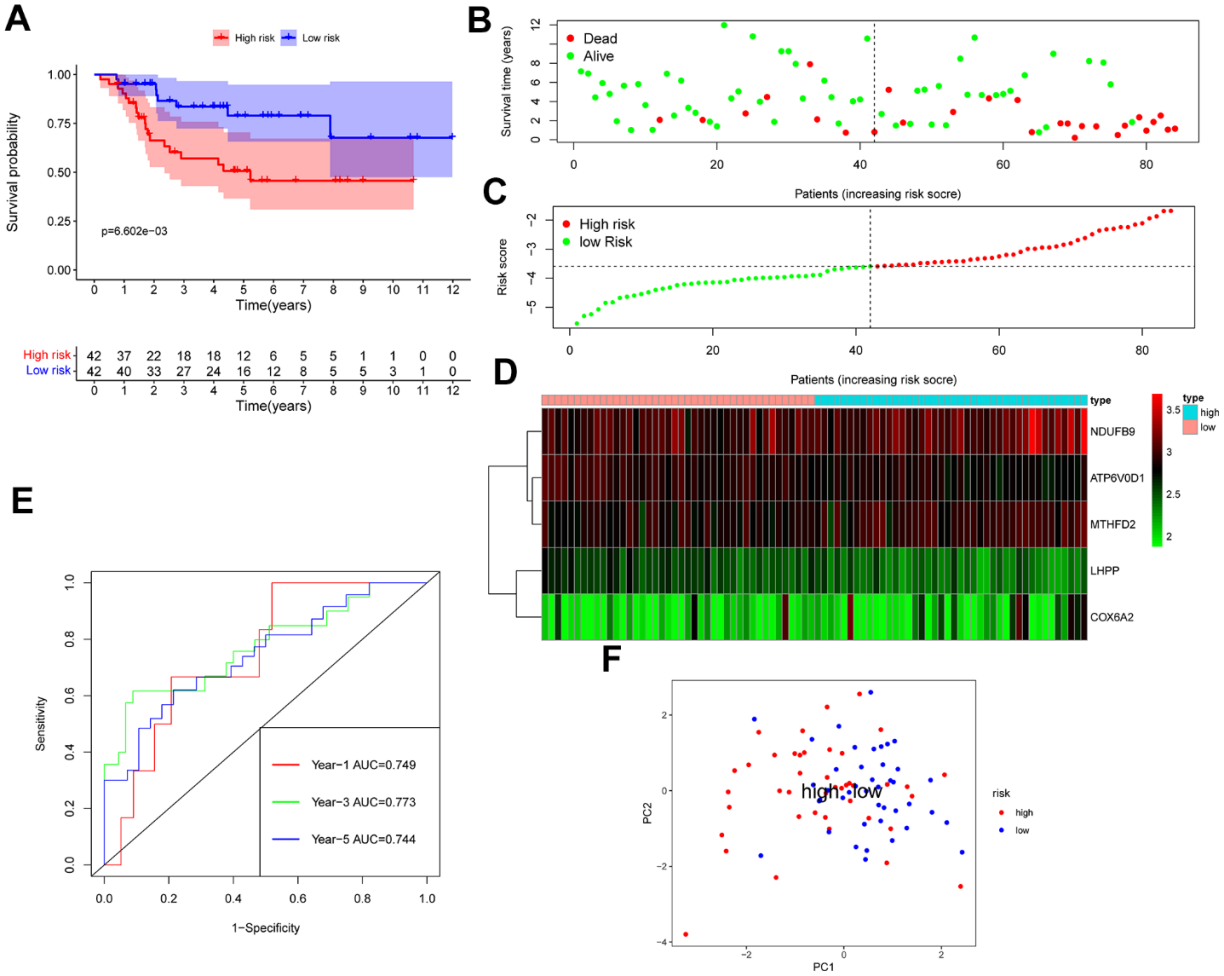
**TCGA osteosarcoma data were used as training groups to validate the effectiveness of the risk score model in predicting survival.** (**A**) Kaplan-Meier survival analysis; (**B**) Scatter chart: green represents survival during follow-up, red represents death during follow-up, abscissa represents risk score, and ordinate represents survival time. (**C**) Risk curve. (**D**) Heat map of gene expression in 84 patients with osteosarcoma. (**E**) Time ROC graph. (**F**) PCA dimensionality reduction analysis chart.

**Figure 4:**
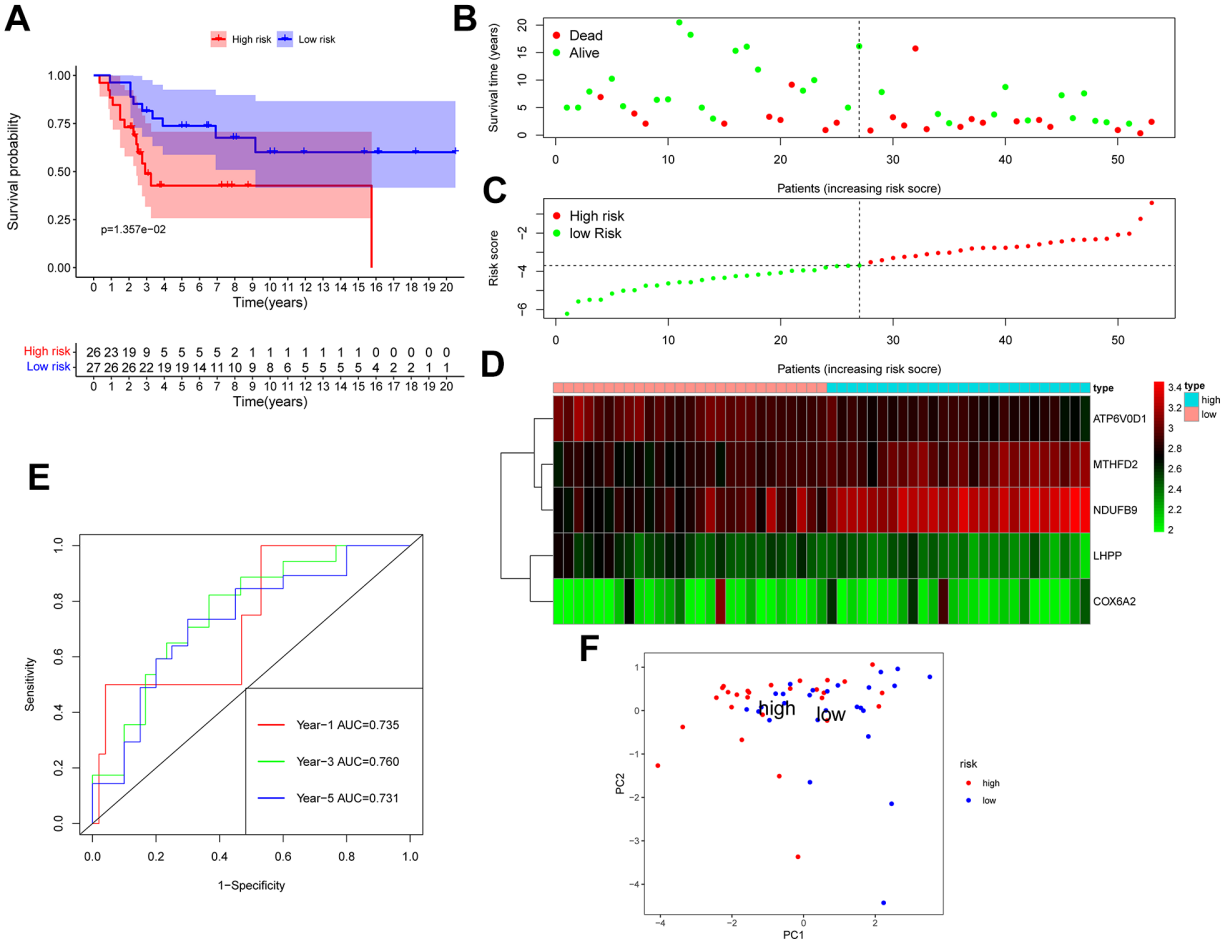
**GSE21257 data was used as a validation group to verify the effectiveness of the risk scoring model in predicting survival.** (**A**) Kaplan-Meier survival analysis; (**B**) Scatter chart: green represents survival during follow-up, red represents death during follow-up, abscissa represents risk score, and ordinate represents survival time. (**C**) Risk curve. (**D**) Heat map of gene expression in 53 patients with osteosarcoma. (**E**) Time ROC graph. (**F**) PCA dimensionality reduction analysis chart.

The GSE28425 dataset was used to obtain 3662 differential genes. The intersection with 342 oxidative phosphorylation-related genes was taken to obtain 68 oxidative phosphorylation-related genes (Figure 1A–1C). A univariate Cox regression analysis was conducted on the training set of individuals to identify five oxidative phosphorylation genes that were associated with prognosis: ATP6V0D1, LHPP, COX6A2, MTHFD2, and NDUFB9 (Figure 2B). Subsequently, the risk-scoring model was developed based on LASSO regression analysis (Figure 2C, 2D). Therefore, the present research computed the risk score of all patients. The correlation coefficients between the five oxidative phosphorylation-related differential expression prognostic genes and risk scores were also examined (Supplementary Figures 1, 2). Analysis of the TCGA training group data demonstrated that individuals with osteosarcoma classified as high-risk exhibited a remarkably poorer prognosis in comparison to those categorized as low-risk (P = 6.602e-03, Figure 3A). Scatter plots and risk curves showed survival and risk scores for all osteosarcoma samples. Individuals with osteosarcoma in the low-risk group demonstrated lower mortality rates and risk coefficients in contrast with the other group (Figure 3B, 3C). Moreover, the heat map highlighted the expression of five oxidative phosphorylation genes related to prognosis in 84 osteosarcoma patients in both groups (Figure 3D). A time-dependent ROC curve was utilized to compute the risk score, yielding respective AUC values of 0.749, 0.773, and 0.744 to predict the OS of individuals suffering from osteosarcoma at 1, 3, and 5 years (Figure 3E). PCA dimensionality reduction analysis showed that the risk-scoring model can better classify osteosarcoma patients into two groups, with considerable variations across the two groups (Figure 3F). The validation set in GSE21257 also demonstrated the effectiveness of the risk model to predict the survival of individuals suffering from osteosarcoma, with low-risk individuals exhibiting a longer OS period in contrast with low-risk individuals (P = 1.357e-02, Figure 4A). Additionally, scatter plots further illustrated that individuals at higher risk demonstrated a more unfavorable prognosis (Figure 4B). Risk curve plots, heat maps, and PCA dimensionality reduction analysis charts showed similar results to the training set (Figure 4C, 4D, 4F). Furthermore, the respective AUC values predicted by the validation group time ROC curve for the OS of individuals suffering from osteosarcoma for 1-, 3-, and 5-year periods were 0.735, 0.760, and 0.731 (Figure 4E). The risk-scoring model demonstrated excellent predictive performance, as evidenced by the AUC values exceeding 0.7 for both validation and training groups.

### Subgroup analysis

The prognosis-predictive efficacy of the risk-scoring model in various clinical feature subgroups was elucidated through the survival analysis of clinical features, with the following findings: Male (*p* = 0.036), female (*p* = 0.076), non-metastatic (*p* = 0.052), age 18 (*p* = 0.068), age 18 (*p* = 0.023), and metastatic (*p* = 0.050) (Supplementary Figure 3A–3F). The validation set application also involved conducting survival analysis based on clinical features, which elucidated the capability of the risk-scoring model to predict outcomes in different clinical feature subgroups (Supplementary Figure 4A–4F). Although *P*-values were not less than 0.05 for many subgroups, it was attributed to the limited sample size of patients and the apparent poorer survival observed among individuals in the high-risk group.

### Tumor microenvironment across high- and low-risk groups

**Figure 5:**
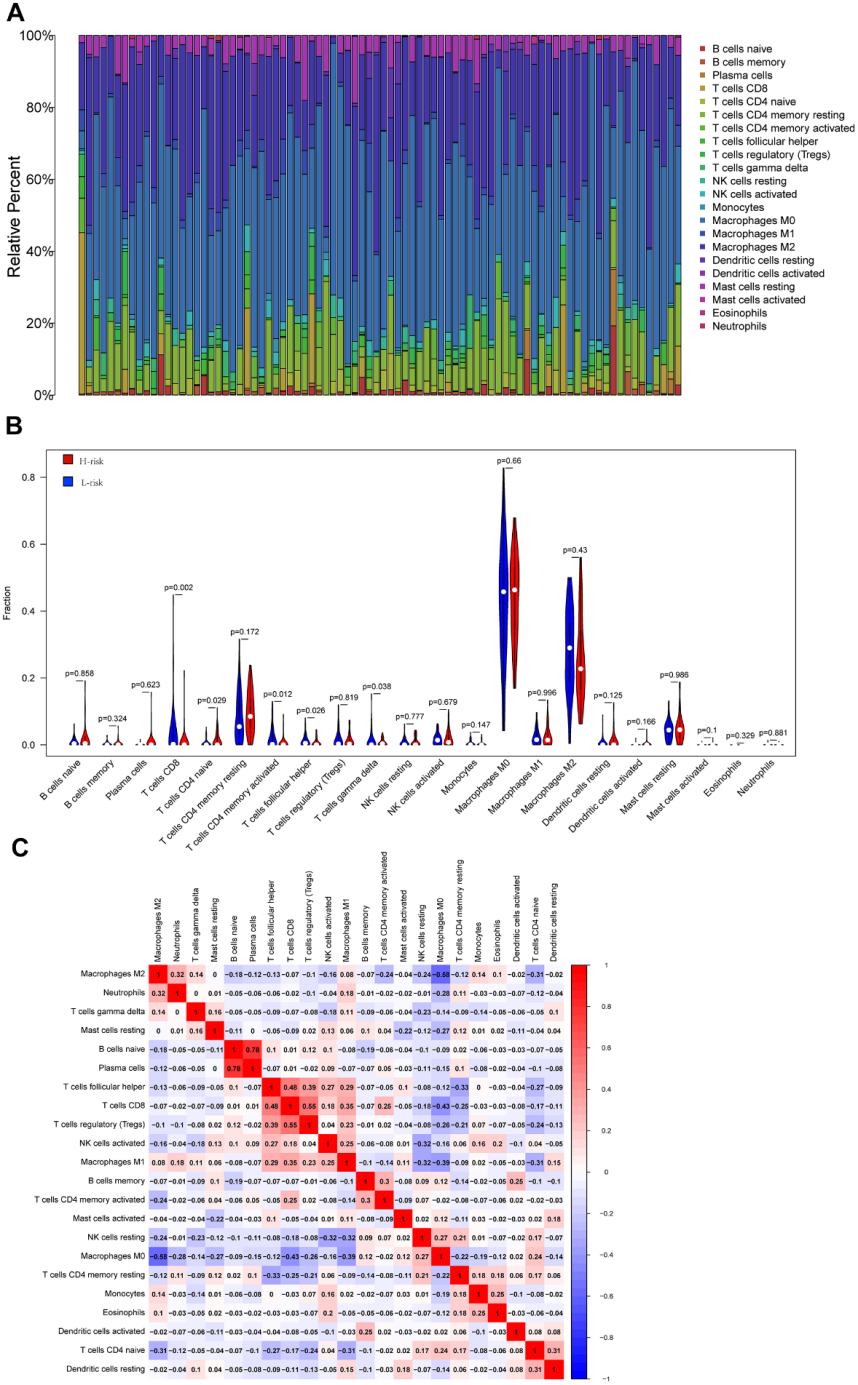
**Cibersort analysis results.** (**A**) The relative proportion of immune cells in 84 osteosarcoma patients; (**B**) The violin diagram shows the difference in the proportion of immune cell infiltration between the low-risk group and the high-risk group. (**C**) Correlation between immune cells.

In order to explore the underlying reasons behind the significant impact of oxidative phosphorylation-related genes on the prognosis of individuals with osteosarcoma, the current research analyzed the results of the infiltration of 22 immune cells across the two risk groups. The results demonstrated the proportion of immune cell composition in 84 patients, highlighting that macrophages accounted for a large proportion of osteosarcoma samples. Macrophages have been found to be substantially involved in the onset and progression of osteosarcoma (Figure 5A). Moreover, this study identified five distinct types of immune cells that exhibited variations across the two groups. In the high-risk group, there was a higher infiltration level of CD4 naive T cells while a lower infiltration level of CD8+T cells, follicular helper T cells, activated CD4 memory T cells, and γ δ T cells was noted (Figure 5B). Subsequently, the link between immune cells was compared, and it was found that M2 macrophages were inversely proportional to most immune cells in osteosarcoma. The correlation coefficient with M0 macrophages was -0.58, showing a high negative correlation, while the correlation coefficient with M1 macrophages was 0.08, showing no correlation. The correlation coefficient between M1 macrophages and M0 macrophages was -0.39. Furthermore, the correlation coefficients for T cells and M1 macrophages were 0.48, 0.55, and 0.35, respectively, and the correlation coefficient for M0 macrophages was -0.43. The ultra-high positive correlation between immature B cells and plasma cells reached 0.78 (Figure 5C).

**Figure 6:**
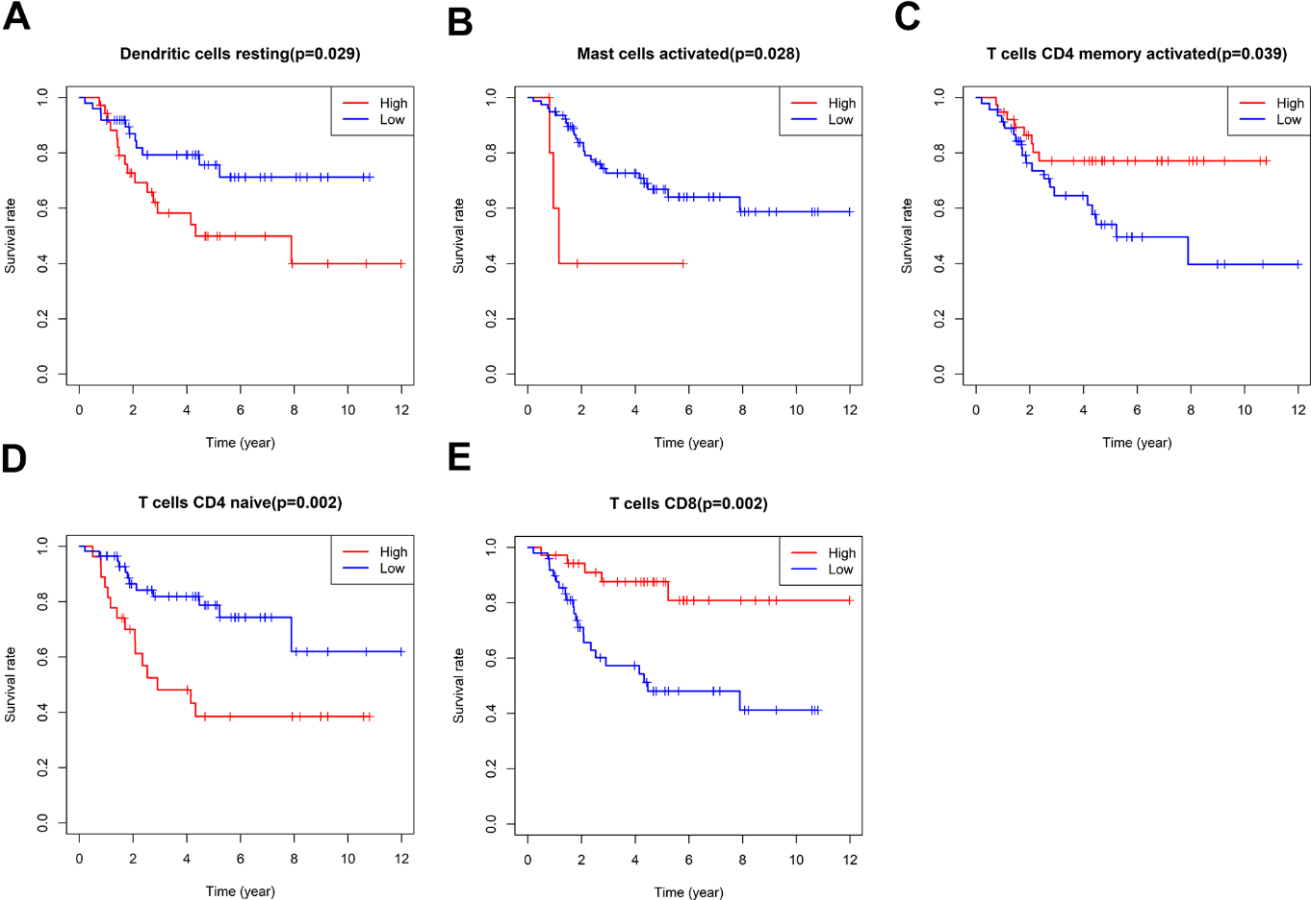
**Survival analysis of immune cells.** The impact of Dendritic cells resting (**A**), Mast cells activated (**B**), T cells CD4 memory activated (**C**), T cells CD4 naïve (**D**) and T cells CD8 (**E**) on the survival of osteosarcoma were examined by employing Kaplan-Meier survival analysis.

According to the relative proportion of 22 immune cells, TCGA osteosarcoma patients were classified into high- and low-risk groups. The impact of various types of immune cells on the survival of osteosarcoma was examined by employing Kaplan-Meier survival analysis. The outcomes highlighted that individuals with a higher proportion of CD8+T cells and activated CD4 memory T cells experienced a more favorable prognosis. On the other hand, patients with a higher proportion of resting dendritic cells, activated mast cells, and immature CD4 T cells demonstrated a poorer prognosis (Figure 6). In addition, the link between oxidative phosphorylation-related differentially expressed genes (DEGs) and immune cells in risk scores and five risk-scoring models was compared. The risk score exhibited a positive link to activated CD4 naive T cells and mast cells while showing an inverse link to activated CD4 memory T cells, CD8+T cells, and follicular helper T cells (Supplementary Figure 5). The expression of the ATP6V0D1 gene was proportional to M1 macrophages, CD8+T cells, and M2 macrophages, and inversely proportional to activated mast cells, M0 macrophages, and CD4 naive T cells (Supplementary Figure 6). Moreover, LHPP gene expression was positively correlated with CD8+T cells and follicular helper T cells, while negatively correlated with CD4 naive T cells (Supplementary Figure 7). The expression of the COX6A2 gene was proportional to naive B cells and inversely proportional to memory B cells (Supplementary Figure 8). MTHFD2 gene expression was not linked to the 22 immune cells examined in this study. NDUFB9 gene expression was proportional to activated mast cells and monocytes (Supplementary Figure 9). Furthermore, the low-risk group exhibited statistically higher matrix, immune, and ESTIMATE scores (matrix score plus immune score). Moreover, the low-risk group exhibited a higher PDCD1LG2 expression, suggesting that this group might be more responsive to PD1 combined with immunosuppressive therapy (Supplementary Figure 10).

### Drugs with potential therapeutic effects on osteosarcoma

In order to further explore why oxidative phosphorylation-related genes in osteosarcoma were significantly correlated with the prognosis of osteosarcoma, this study conducted a drug sensitivity analysis of osteosarcoma. Drug sensitivity analysis showed that Bortezomib (*p* = 1.3e − 06), CGP. 082996 (*p* = 7.7e − 05), GNF. 2 (*p* = 0.00013), MG.132 (*p* = 3.2e − 06), NVP.TAE684 (*p* = 1.8e − 05), PAC.1 (*p* = 0.00018), PF.02341066 (coxotinib) (*p* = 3.4e − 06), and Roscovitine (*p* = 2.5e − 05) demonstrated substantial sensitivity in both risk groups. Individuals in the low-risk group displayed more sensitivity to Bortezomib, CGP. 082996, GNF. 2, MG. 132, NVP. TAE684, F.02341066, and Roscovitine. In contrast, the individuals at higher risk exhibited greater sensitivity to PAC (Supplementary Figure 11). The stability of proteins that hinder cell survival and cell cycle advancement, such as p53, was increased by bortezomib. CGP60474 is a protein kinase C (PKC) inhibitor, and GNF-2 is a Bcr-Abl fusion gene inhibitor. MG-132 is an inverse proteasome inhibitor with the ability to induce tumor cell apoptosis. Moreover, NVP-TAE684 is a selective inhibitor of anaplastic lymphoma kinase (ALK), which is associated with the pathogenesis of various cancers and can serve as an important therapeutic target. PAC-1 drugs have the ability to induce cell apoptosis, and their use alone or in combination with chemotherapy has shown anticancer effects in lung cancer, melanoma, and osteosarcoma, among others. Furthermore, PF-02341066 was an ALK inhibitor that can induce autophagy in various tumor cell lines by inhibiting the STAT3 pathway. Roscovitine was a selective inhibitor of cyclin-dependent kinase (CDK). CDK is an essential cell cycle regulator and often participates in the deregulation of human tumors.

### Nomogram of the prediction model

**Figure 7:**
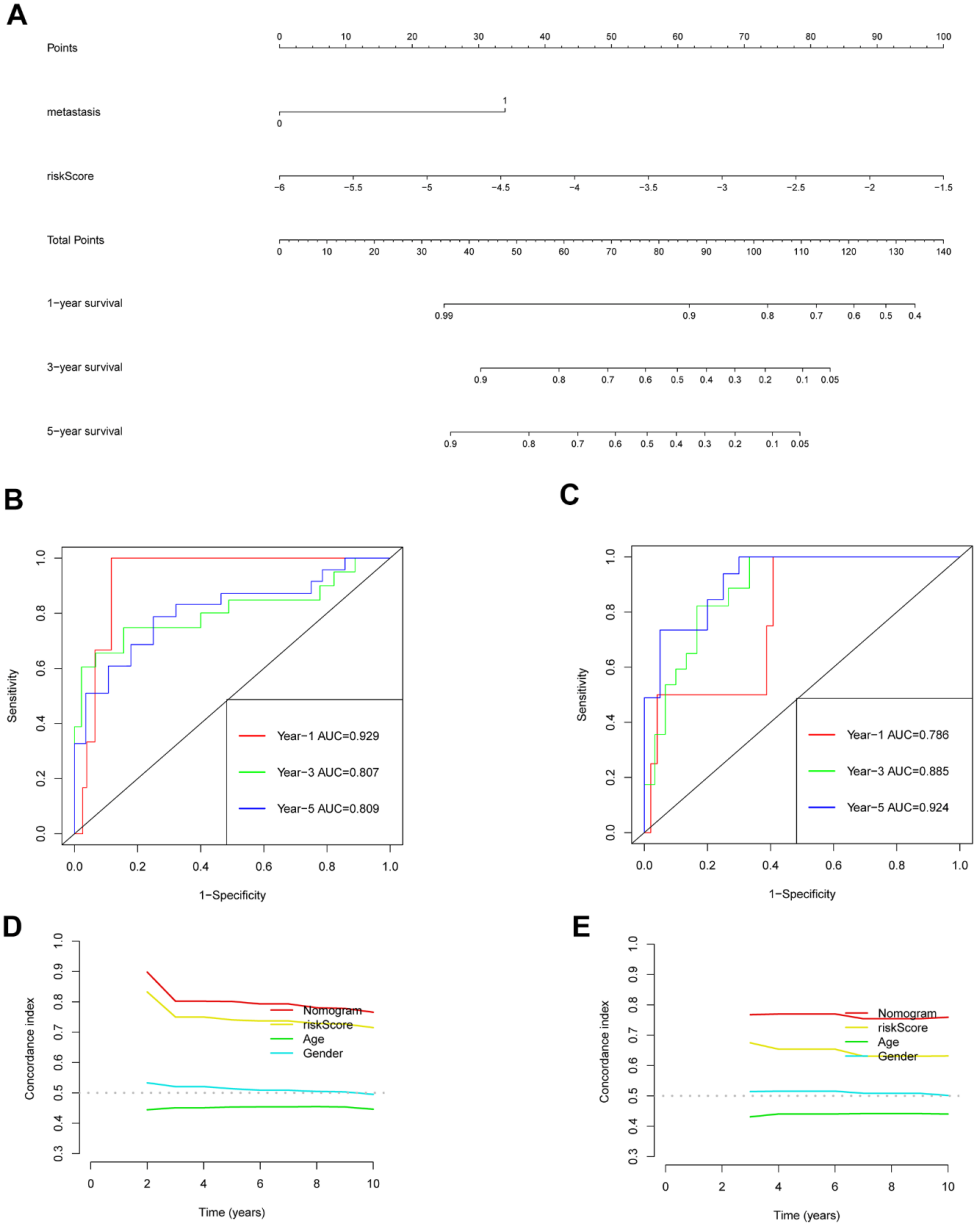
**Validation of the prognostic model nomogram.** (**A**) Nomogram. (**B**) Time ROC curve of training set. (**C**) Time ROC curve of the validation set. (**D**) The C-index calibration curve for age, gender, risk score, and nomogram of the training set. (**E**) Validate the C-index calibration curve for age, gender, risk score, and nomogram.

Univariate and multivariate Cox regression analyses were conducted to explore the link between gender, age, metastasis, risk-scoring, and prognosis of individuals who suffer from osteosarcoma (Supplementary Figure 12A, 12B). The findings demonstrated that both metastasis and risk score were identified as independent predictors of prognoses for individuals suffering from osteosarcoma. The 1-, 3-, and 5-year OS of the patients were predicted using a nomogram that took the outcomes of multivariate Cox regression into account (Figure 7A). This nomogram included the risk-scoring index of the patient and whether the patient had metastasis. The predicted performance results of the nomogram estimated by the ROC curve highlighted that the respective AUC values of 1-, 3-, and 5-year OS of the training set were 0.929, 0.807, and 0.809 (Figure 7B). However, the respective AUC values of 1-, 3- and 5-year OS of the verification set were 0.786, 0.885, and 0.924 (Figure 7C). The C index also highlighted a good predictive performance exhibited by the prognosis model (Figure 7D, 7E).

### Gene features set enrichment analysis

To investigate the possible mechanism underlying the association between oxidative phosphorylation-related genes and the prognosis of individuals with osteosarcoma, an enrichment analysis of DEGs (|log2FC|>1, *p*-value<0.05) was carried out across the two risk groups. GO enrichment analysis revealed that the DEGs were primarily associated with biological processes related to myofibril assembly and muscle contraction. In terms of cellular components, the enrichment was predominantly observed in myofibrils. Furthermore, the molecular functions of these genes were found to be primarily associated with muscle structural components. The pathways for KEGG enrichment analysis included neural active ligand- receptor interaction pathways. GSEA enrichment analysis highlighted that the enrichment pathways of DEGs between both risk groups included leukocyte adhesion, cell activation, actin-mediated cell contraction, and positive regulation of external stimulus responses (Supplementary Figure 13).

## DISCUSSION

Osteosarcoma seriously affects the health of adolescents and can be metastasized to the lung and other tissues in the early stage [11]. Numerous reports have confirmed the significant role of oxidative phosphorylation in the malignant progression of tumors. Additionally, oxidative phosphorylation is considered to be the key mechanism of energy metabolism in osteosarcoma [2, 10, 12]. Therefore, investigating oxidative phosphorylation-related genes in osteosarcoma becomes crucial for predicting the prognosis of patients and identifying potential therapeutic targets. In this research, five oxidative phosphorylation genes linked to the prognosis of individuals with osteosarcoma were screened out, including ATP6V0D1, LHPP, COX6A2, MTHFD2, and NDUFB9. Among them, COX6A2, MTHFD2, and NDUFB9 were positively correlated with risk scores, suggesting that they were risk genes for unfavorable prognosis among individuals with osteosarcoma. On the other hand, ATP6V0D1 and LHPP were negatively correlated with risk scores, which suggested that they are protective genes for the clinical prognosis of patients with osteosarcoma.

In eukaryotic cells, vacuolar ATPase (V-ATPase), which was encoded by ATP6V0D1, is in charge of acidifying a number of intracellular compartments. As a result, the majority of the energy needed for vacuolar system transport operations is provided by V-ATPase [13]. The findings from the current research align with the results of two prior studies, both of which have demonstrated that ATP6V0D1 is a protective gene for osteosarcoma [13, 14]. The current investigation found no molecular biology studies related to ATP6V0D1 and osteosarcoma cells, suggesting that ATP6V0D1 may be a new research direction [15]. The hydrolase encoded by LHPP exhibits a wide range of substrate specificity and is capable of hydrolyzing inorganic diphosphate as well, albeit with relatively lower efficiency [16]. To date, there have been no studies exploring the association of LHPP with osteosarcoma. However, the present study has presented novel findings, establishing LHPP as a prognosis-protective gene for osteosarcoma. This discovery holds immense significance and opens up new avenues for further research in this field [17]. Moreover, LHPP is an oncogene in colorectal, pancreatic, bladder, thyroid, and prostate cancers [18–22]. Cytochrome c oxidase, which is the final enzyme in the mitochondrial electron transport chain, catalyzes the reduction of oxygen to water as well as oxidative phosphorylation [23]. The oxidative respiratory chain consists of three complex, multisubunit groups - Complex II (CII), Complex III (CIII) also referred to as cytochrome b-c1 complex, and Complex IV (CIV) or cytochrome c oxidase. These complexes work together to transfer electrons from NADH and succinate to molecular oxygen, leading to the formation of an electrochemical gradient across the inner mitochondrial membrane. Subsequently, this gradient drives transmembrane transport and facilitates the functioning of ATP synthase [10, 24]. There are also no relevant studies on COX6A2 and the prognosis of osteosarcoma. Moreover, there is only one paper on COX6A2 and cancer prognosis, which demonstrated that COX6A2 is a prognostic protective gene in esophageal cancer [25]. MTHFD2 is responsible for encoding a mitochondrial bifunctional enzyme that performs both methylenetetrahydrofolate dehydrogenase and methyltetrahydrofolate cyclohydrolase activities and is encoded by nuclear DNA [26]. The homodimeric nature of the action of the enzyme and its particular need for magnesium and inorganic phosphate distinguishes it from other enzymes [27]. The finding that MTHFD2 is a prognostic risk gene for osteosarcoma was also demonstrated for the first time in this study. Among other types of cancers, MTHFD2 has been identified as a risk gene in ovarian, colorectal, lung, and breast cancers [26, 28–32]. A subunit of the mitochondrial oxidative phosphorylation complex I (nicotinamide adenine dinucleotide: ubiquinone oxidoreductase) is encoded by the gene NDUFB9 [33]. Complex I is situated in the inner membrane of mitochondria and is responsible for oxidizing nicotinamide adenine dinucleotide and transferring electrons to coenzyme Q. Malfunctions in complex I are the primary cause of oxidative phosphorylation disorders and are linked to various illnesses. Furthermore, the current study also highlighted the function of NDUFB9 as a prognostic risk gene for osteosarcoma [33, 34]. However, in breast cancer, NDUFB9 serves as a prognostic protective gene [35], exhibiting inconsistency with the outcomes of the current investigation.

While developing and validating the prognosis model, it was found that the expression levels of oxidative phosphorylation-related genes varied among osteosarcoma patients and influenced their immunity status. Patients with greater immune activity had a more favorable prognosis than those with lower immune activity. The present research highlighted that increased T cells CD4 naive, and reduced T cells CD8, T cells CD4 memory activated, T cells follicular helper, and T cells gamma delta were found in the high-risk group in comparison with the low-risk group. The current investigation also indicated that oxidative phosphorylation has an impact on the immune status of individuals suffering from osteosarcoma, which in turn affects the development of the disease [36]. Although further investigation is needed to elucidate the precise mechanism underlying this study, its potential to offer novel perspectives into the molecular mechanisms responsible for the onset and progression of osteosarcoma is significant. Additionally, the current research could aid in exploring targeted therapies for osteosarcoma, which could be particularly beneficial for patients who show resistance to conventional chemoradiotherapy. Overall, this study could have significant clinical implications [37, 38].

This study is subject to certain limitations, including the relatively small sample sizes of both the training and validation cohorts. Therefore, further verification of the prognostic model is required using a larger cohort.

## CONCLUSIONS

The present study established a risk-scoring model incorporating five oxidative phosphorylation genes that were linked to the prognosis of osteosarcoma. Additionally, a nomogram for the prediction of the OS rate of osteosarcoma patients was developed and validated. This model exhibits good accuracy and universality, which makes it a valuable tool for predicting the clinical outcome of patients with osteosarcoma. Moreover, its ability to provide reliable predictions can serve as a valuable reference for clinical treatment decisions. At the same time, the study also found that the prognosis based on these five genes is congruent with the impact of immune cells on the prognosis of osteosarcoma, suggesting that these genes might be linked to immune cells. The findings of this study offer new tools for orthopedic surgeons to improve their clinical decision-making process in the treatment of osteosarcoma. Additionally, the identification of new therapeutic targets in osteosarcoma may offer improved efficacy and outcomes for these patients.

## MATERIALS AND METHODS

### Data source and preprocessing

Osteosarcoma transcriptome and clinical data were retrieved from the UCSC Xena (http://xena.ucsc.edu/) as the training set. A validation set for this study was obtained by acquiring transcriptome and clinical data of individuals diagnosed with osteosarcoma from the GSE21257 datasets. The datasets were retrieved from the publicly accessible GEO (Gene Expression Omnibus) database. Researchers can access these datasets by following the provided link: https://www.ncbi.nlm.nih.gov/geo. The GSE28425 dataset was used to screen for osteosarcoma differential genes.

### Development of the risk-scoring model

Using the oxidative phosphorylation-related genes screened out above, the model was constructed based on the training set data. Differential genes above 0.5-fold were screened using GSE28425, whereas univariate Cox regression analysis was conducted to screen prognostic oxidative phosphorylation-related genes. Subsequently, LASSO regression analysis was conducted to calculate the risk coefficient (coefi) of all oxidative phosphorylation-related genes. To compute the risk score for every individual, the expression (expi) and coefficient (coefi) values of all oxidative phosphorylation genes related to prognosis were added. This was done for every sample in the study, and the resulting value was considered as the risk score of the patient (risk score = (coefi × expri)). By utilizing the median value of risk scores, individuals were classified into high- and low-risk groups. In order to examine whether a noteworthy disparity in the overall survival of individuals between both groups existed, a Kaplan-Meier survival analysis was conducted. The capability of the risk scoring model for making predictions was analyzed by calculating the area under the curve (AUC) using receiver operating characteristic (ROC) curve analysis.

### Analysis of subgroups and confirmation of the capacity of the risk prognostic model for independent prognostication

Patient subgroups were created based on age (<18, ≥ 18), sex (male, female), and if metastasis had taken place (metastatic or non-metastatic). To ascertain whether there were appreciable variations in OS across high- and low-risk patients in each subgroup, Kaplan-Meier survival analysis was utilized.

### Analysis of tumor microenvironment across the two risk groups

The abundance of 22 types of immune cells in both risk groups (high and low) was calculated by utilizing the CIBERSORT algorithm (https://cibersortx.stanford.edu/). Additionally, high and low groups were established as per the median expression of different immune cells, and the difference in survival time across the two groups was determined. The ESTIMATE (Estimation of Stromal and Immunological Cells in Malignant Tumor Tissues using Expression Data) algorithm was employed to assess the stromal, immunological, and ESTIMA scores, as well as the tumor purity of various molecular subgroups. Ultimately, the association of genes with immune cells was examined.

### Drug sensitivity analysis

Drug sensitivity analysis was conducted using “Rs” “limma”, “ggpubr”, and “pRRophic” packages to examine the varying sensitivities of drugs for patients in both risk groups in the bone and meat data of TCGA. Moreover, the potential osteosarcoma treatment drugs were screened with a screening criterion of *p*-value < 0.001.

### Nomogram of the prediction model

According to the clinical information of sex, age, metastasis, and risk score of the oxidative phosphorylation gene, univariate and multivariate Cox regressions were implemented, and the nomogram was constructed according to the results. Following the calculation of OS for all patients, their 1-year, 3-year, and 5-year survival rates were predicted, and a ROC curve was developed to measure the predictive accuracy of the nomogram.

### Gene set enrichment analysis

In addition, enrichment analyses (GSEA, GO, and KEGG) were conducted on the differentially expressed genes (DEGs) identified between both risk groups.

### Statistical analysis

R (v 4.2.0) was employed to carry out all statistical analyses. OS curves were generated by utilizing the Kaplan-Meier method and assessed by means of the log-rank test. A significance level of 0.05 or greater was used for all two-tailed *p*-values.

### Data availability statement

The data that support the findings of this study are openly available in Therapeutically Applicable Research to Generate Effective (TARGET) database and GEO (Gene Expression Omnibus).

## Supplementary Material

Supplementary Figures
