# Supplementary material for: Construction of an oxidative phosphorylation-related gene signature for predicting prognosis and identifying immune infiltration in osteosarcoma
Source: Aging (Albany NY). 2024 Mar 20;16(6):5311–35. doi: 10.18632/aging.205650 (PMC11006489; doi:10.18632/aging.205650)
Supplement: Supplementary Figures [file aging-16-205650-s001.pdf]

SUPPLEMENTARY FIGURES

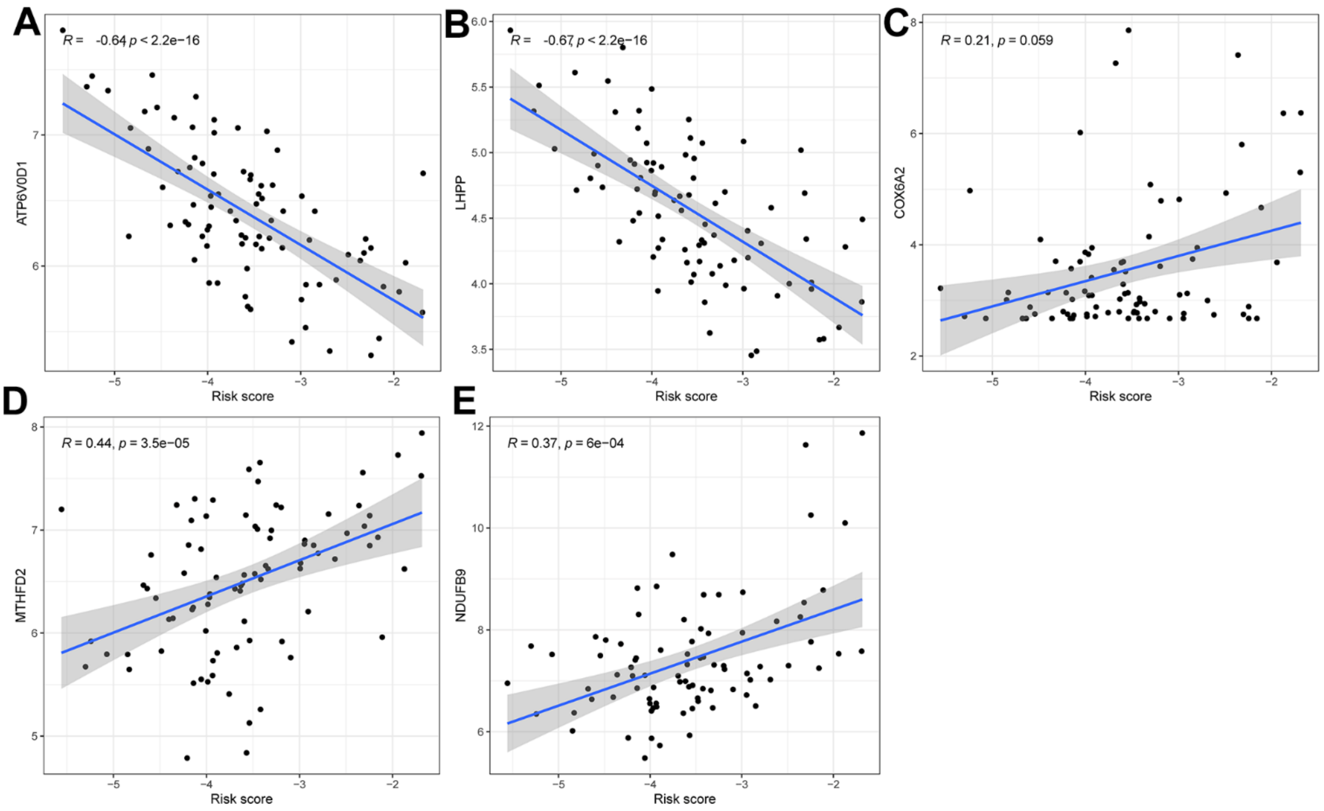

Supplementary Figure 1. Correlation analysis between 5 genes and risk score in TARGET data. (**A**–**E**) The correlation coefficients between ATP6V0D1, LHPP, COX6A2, MTHFD2, and NDUFB9 in TARGET osteosarcoma data and risk scores, respectively.

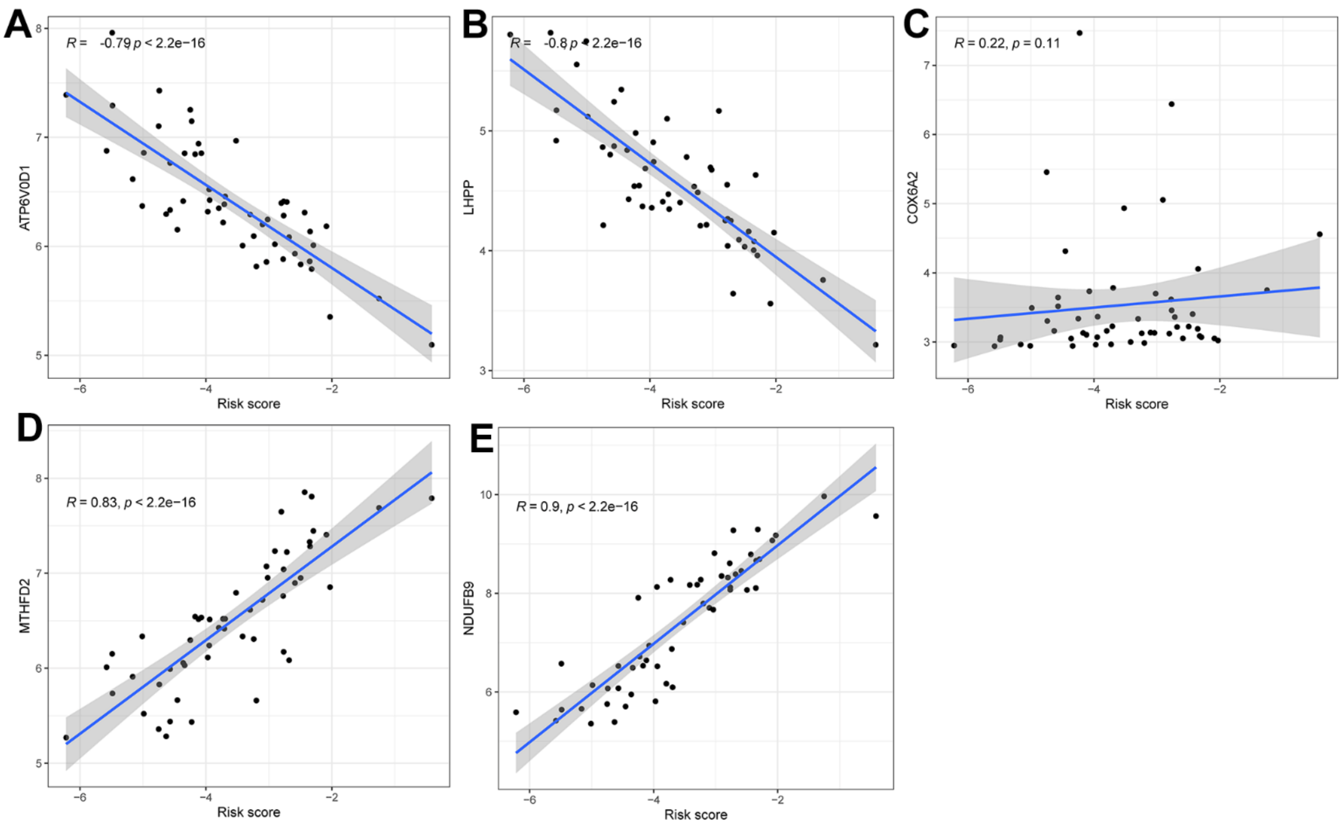

Supplementary Figure 2. Correlation analysis between 5 genes and risk score in GSE21257 dataset. (**A**–**E**) The correlation coefficients between ATP6V0D1, LHPP, COX6A2, MTHFD2, and NDUFB9 in the GSE21257 dataset and risk scores, respectively.

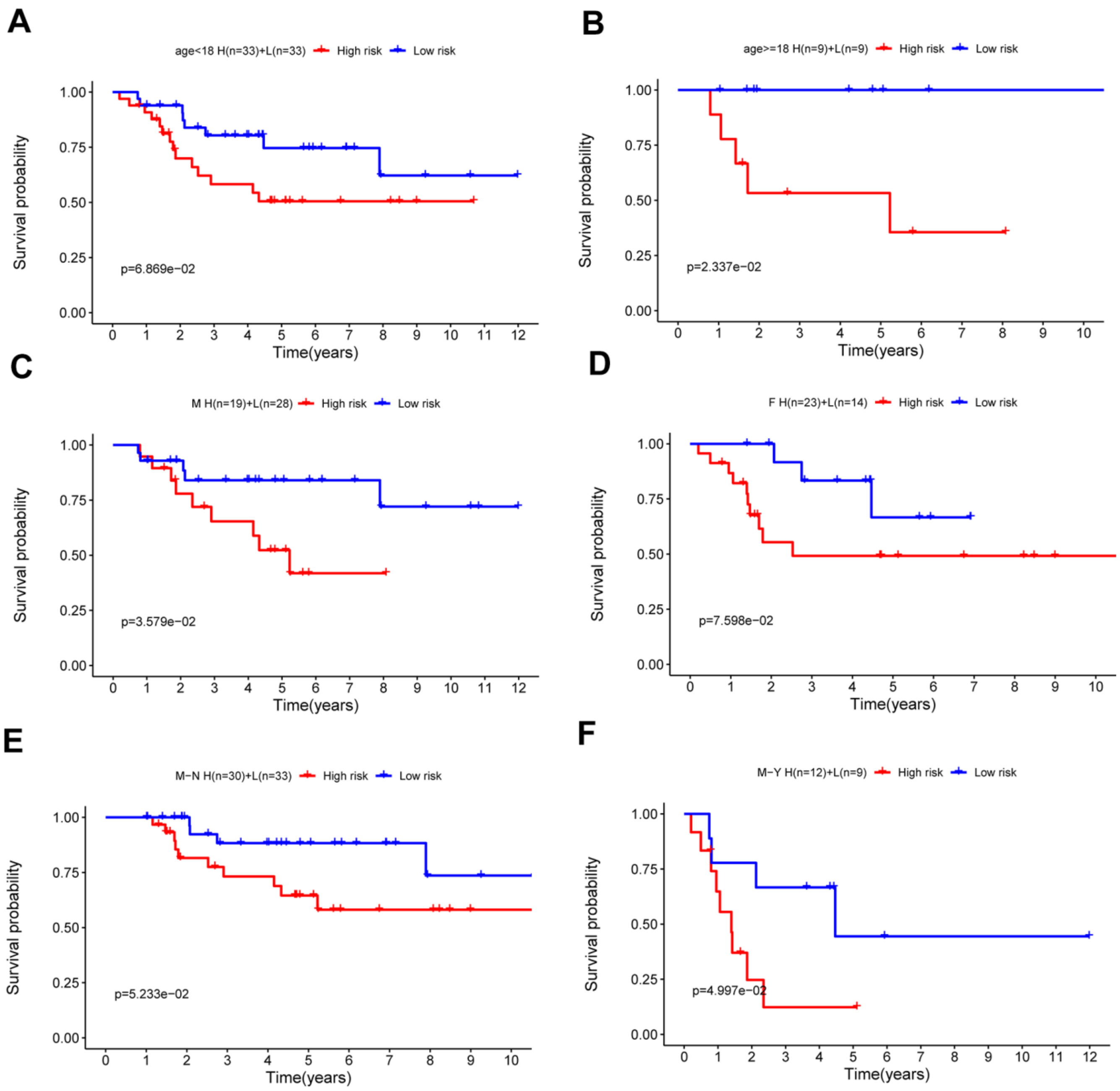

Supplementary Figure 3. TCGA clinical subgroup survival analysis of the risk score of osteosarcoma oxidative phosphorylation genes. The results of the risk-scoring model in the training group were <18 (**A**) male (**B**) nonmetastatic (**C**) ≥18 (**D**) female (**E**) and metastatic (**F**) subgroups.

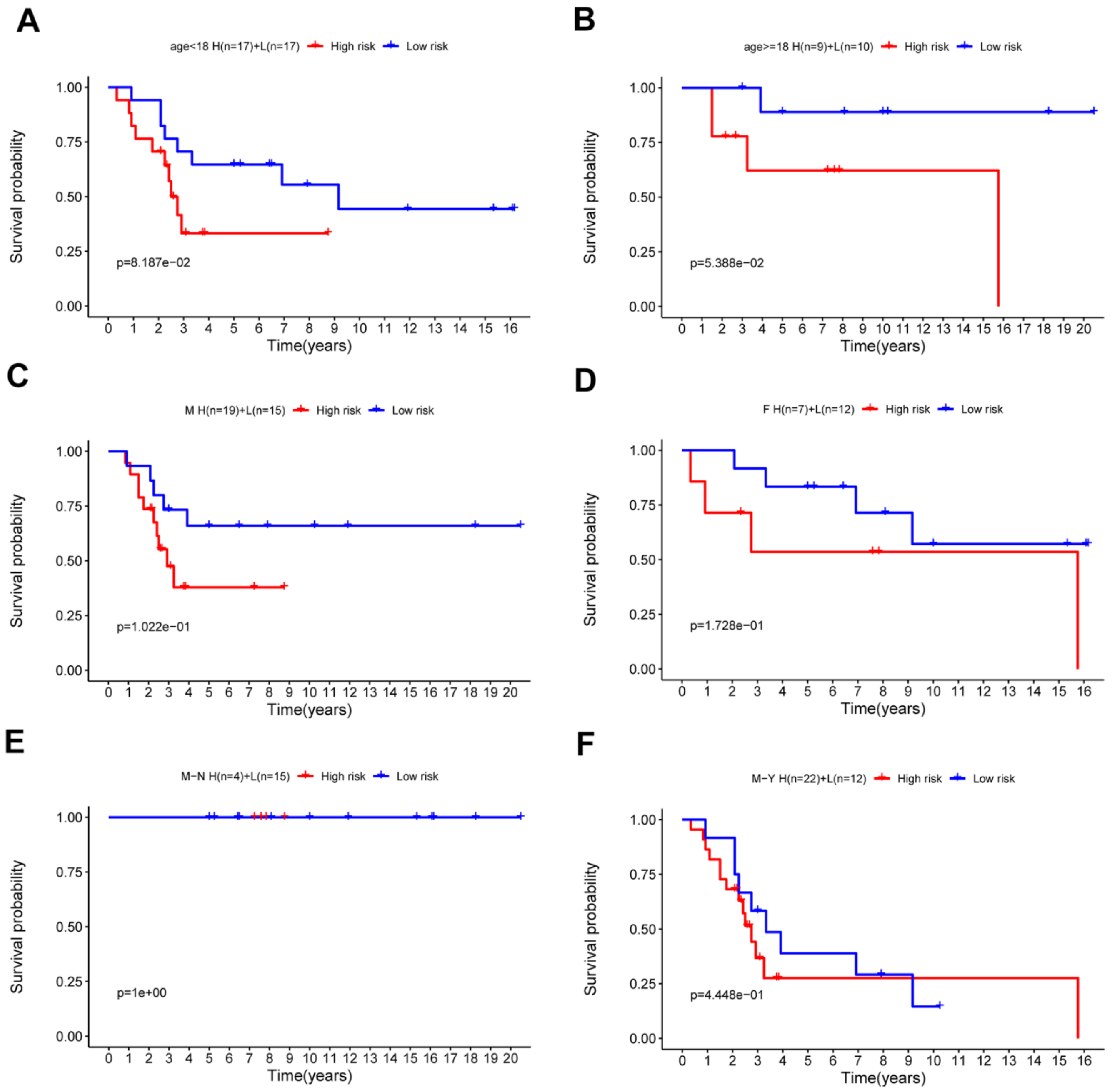

Supplementary Figure 4. GSE21257 clinical subgroup survival analysis of the risk score of osteosarcoma oxidative phosphorylation genes. The results of the risk-scoring model in the validation group were <18 (**A**), male (**B**), nonmetastatic (**C**), ≥18 (**D**), female (**E**), and metastatic (**F**) subgroups.

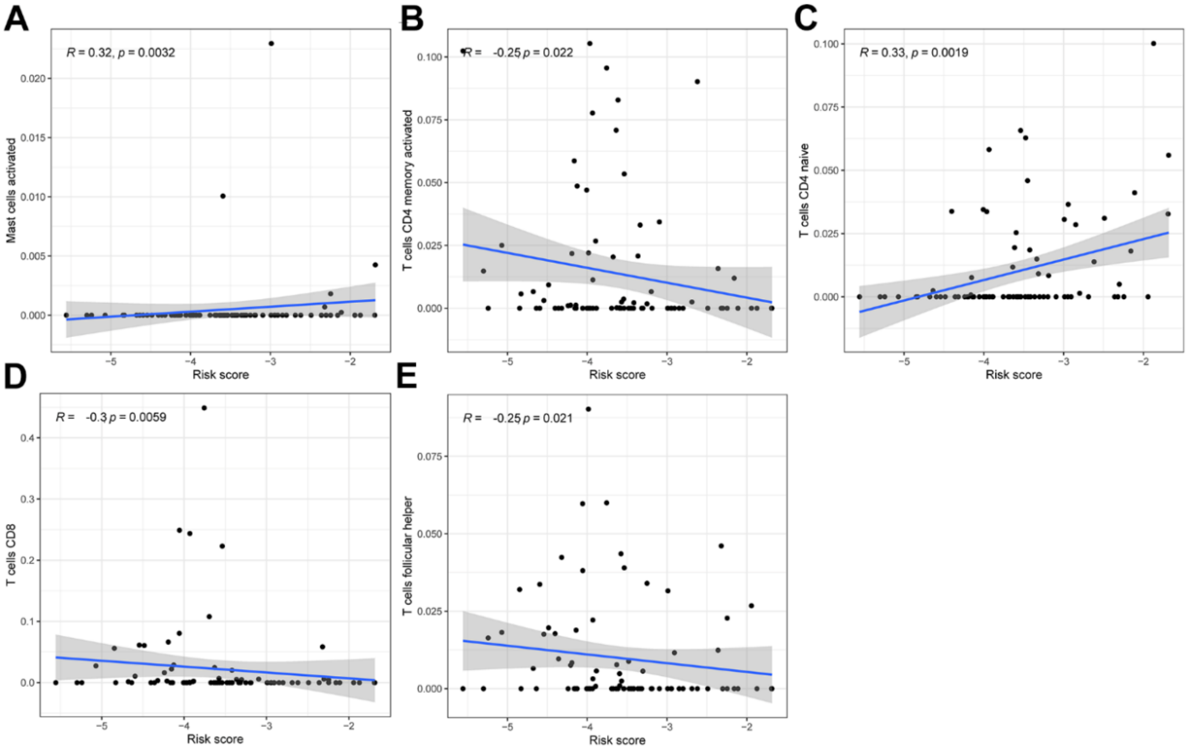

Supplementary Figure 5. Correlation analysis between risk score and immune cells. (**A**–**E**) The correlation coefficients between risk score and Mast cells activated, T cells CD4 memory activated, T cells CD4 naïve, T cells CD8 and T cells follicular helper, respectively.

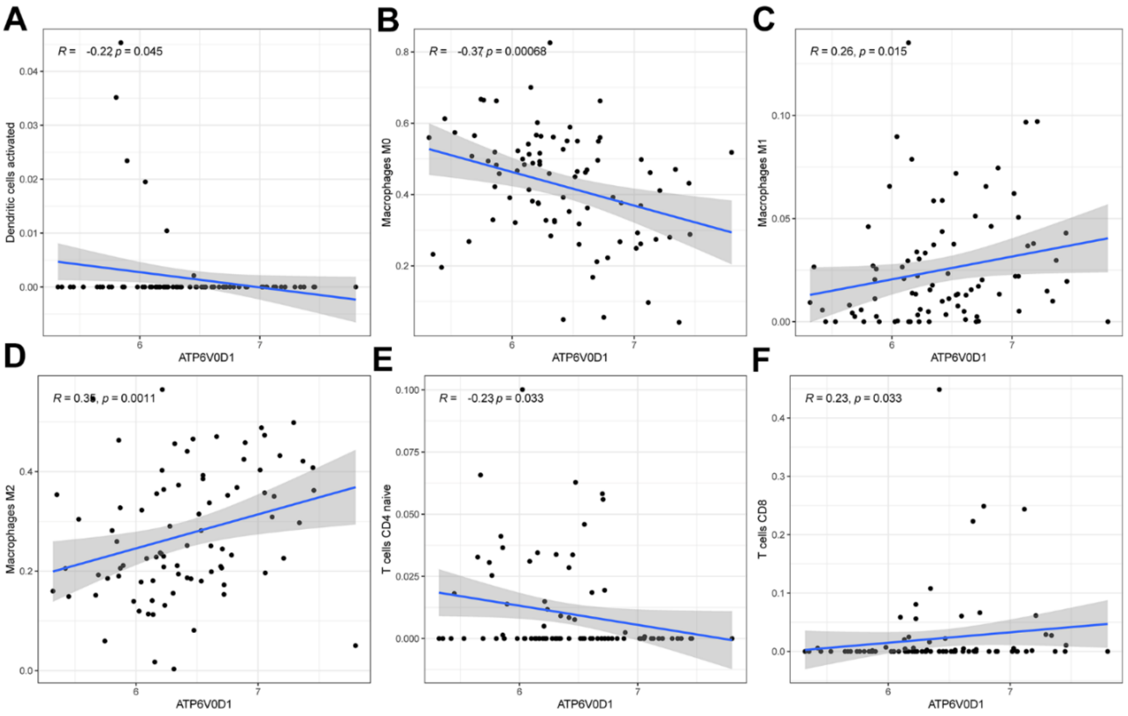

Supplementary Figure 6. Correlation analysis between ATP6V0D1 gene expression and immune cells. (**A**–**F**) The correlation coefficients between ATP6V0D1 and Dendritic cells activated, Macrophages M0, Macrophages M1, Macrophages M2, T cells CD4 naïve and T cells CD8, respectively.

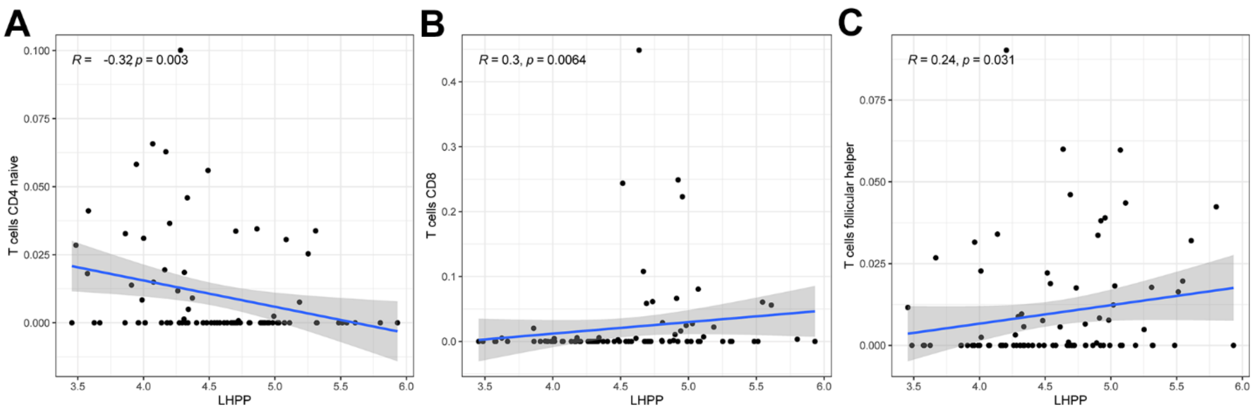

Supplementary Figure 7. Correlation analysis between LHPP gene expression and immune cells. (**A**–**C**) The correlation coefficients between LHPP and T cells CD4 naïve, T cells CD8 and T cells follicular helper, respectively.

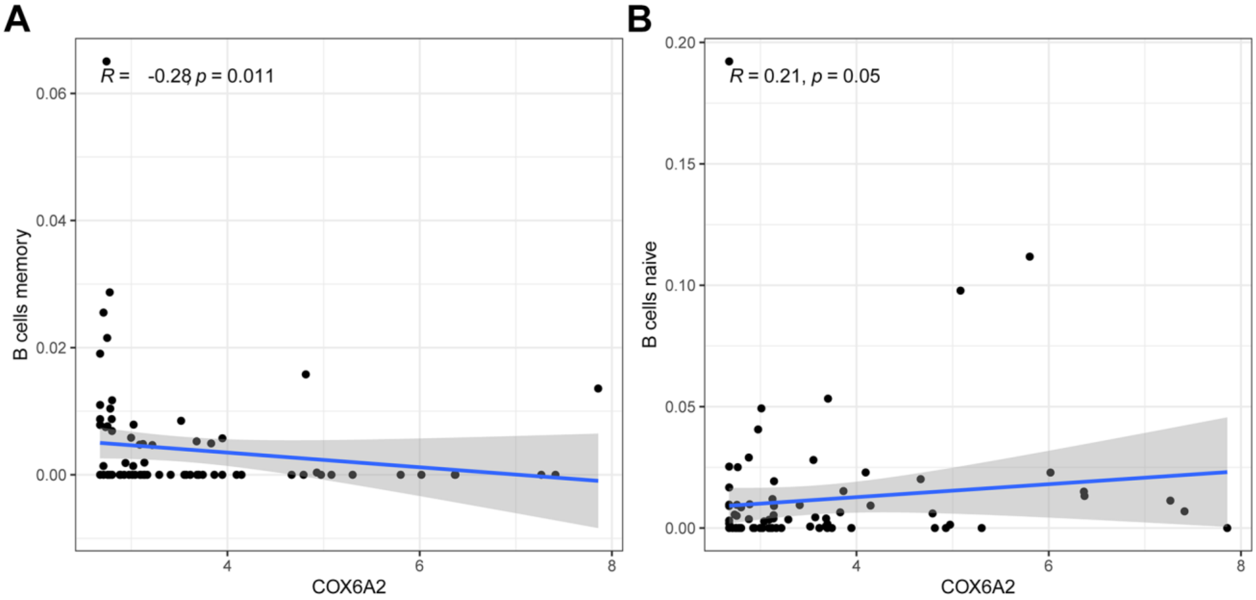

Supplementary Figure 8. Correlation analysis between COX6A2 gene expression and immune cells. (**A**, **B**) The correlation coefficients between COX6A2 and B cells memory and B cells naive, respectively.

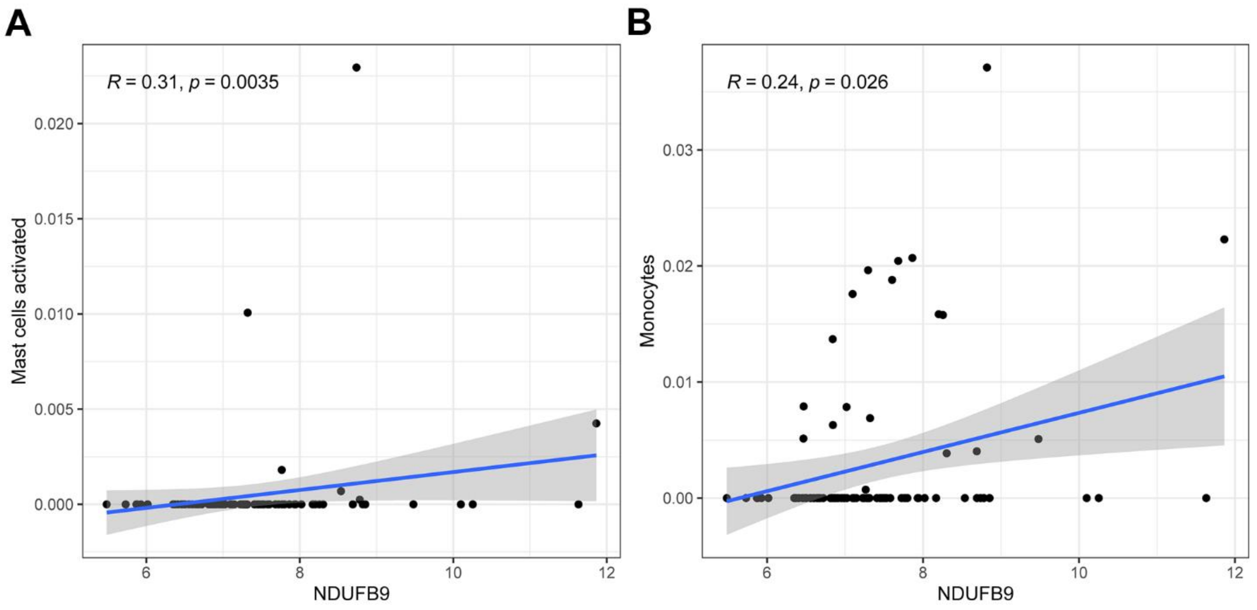

Supplementary Figure 9. Correlation analysis between NDUFB9 gene expression and immune cells. (**A**, **B**) The correlation coefficients between NDUFB9 and Mast cells activated and Monocytes, respectively.

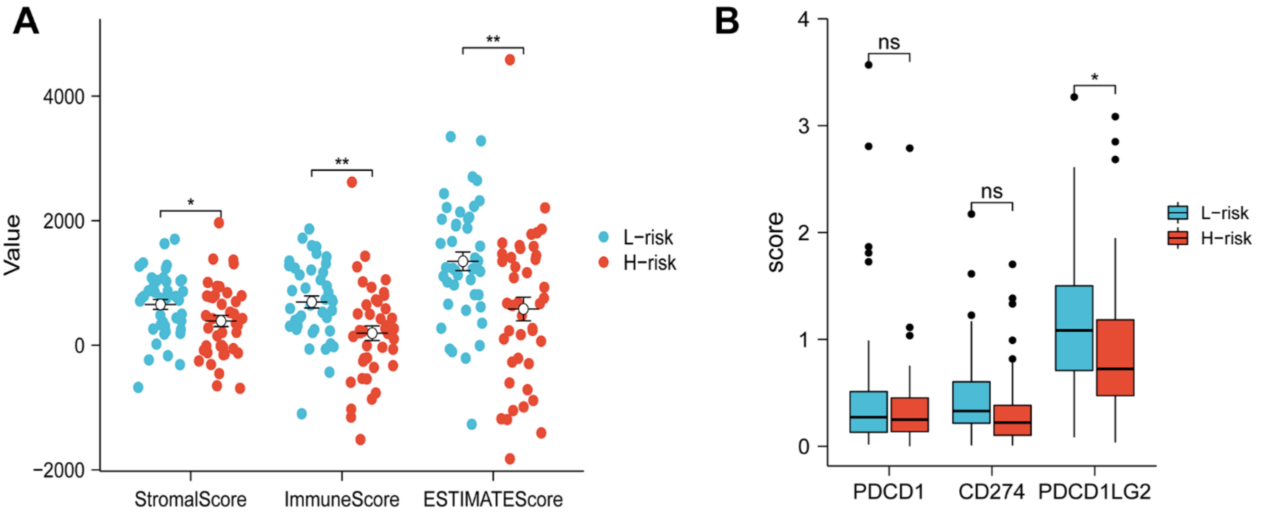

Supplementary Figure 10. Results of immunity analysis. (**A**) Differences in matrix scores, immune scores, and ESTIMATE scores between the low-risk group and the high-risk group. (**B**) The difference in the expression of PDCD1, CD274, and PDCD1LG2 between the low-risk group and the high-risk group. (*, p <0.05, **, p <0.01, ***, p <0.001).

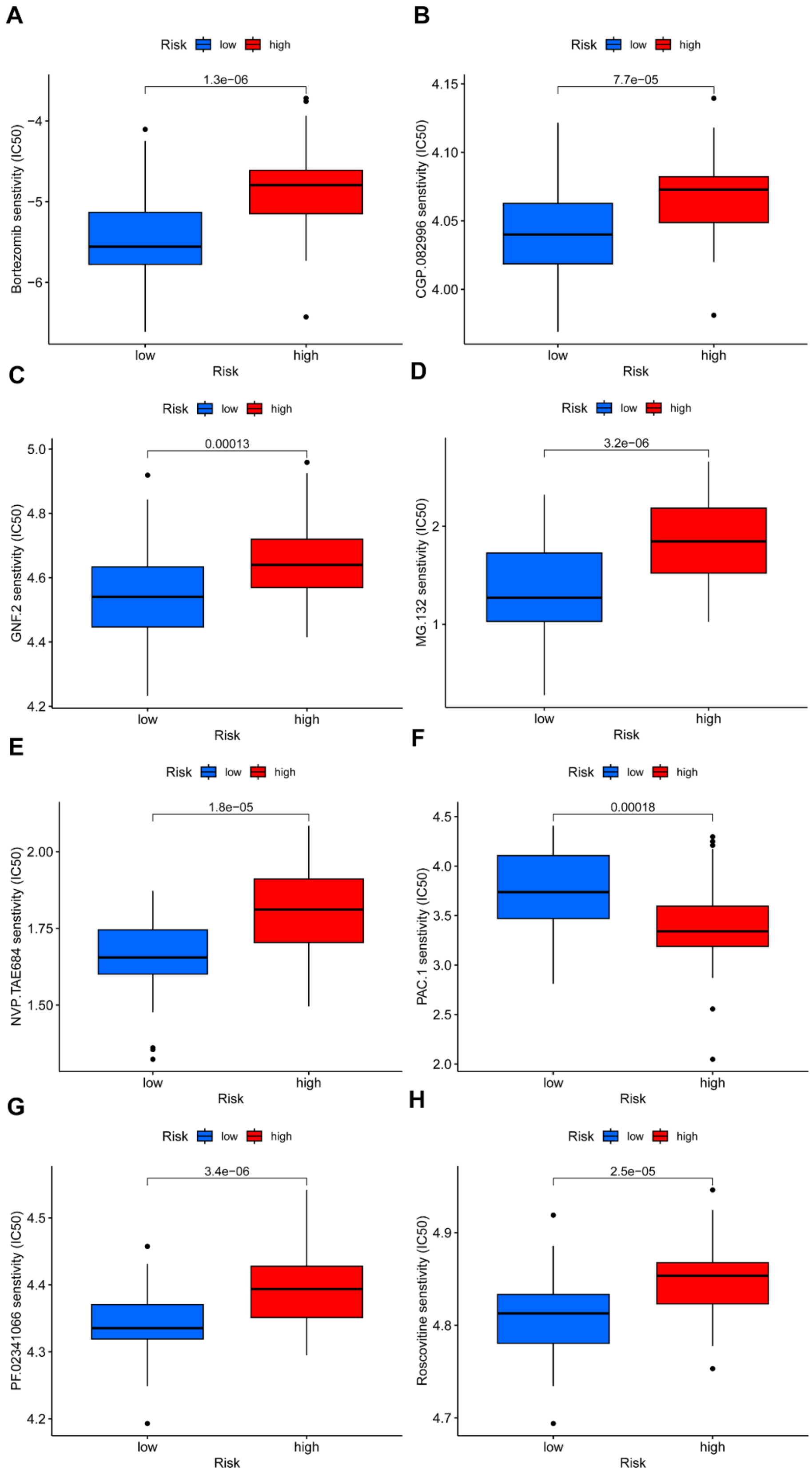

Supplementary Figure 11. Results of drug sensitivity analysis. (**A**) Bortezomib (*p* =1.3e – 06). (**B**) CGP.082996 (*p* = 7.7e−05). (**C**) GNF.2 (*p* = 0.00013). (**D**) MG.132 (*p* = 3.2e−06). (**E**) NVP.TAE684 (*p* = 1.8e−05). (**F**) PAC.1 (*p* = 0.00018). (**G**) PF. 02341066 (clozotinib) (*p* =3.4e – 06). (**H**) Roscovitine (*p* = 2.5e−05).

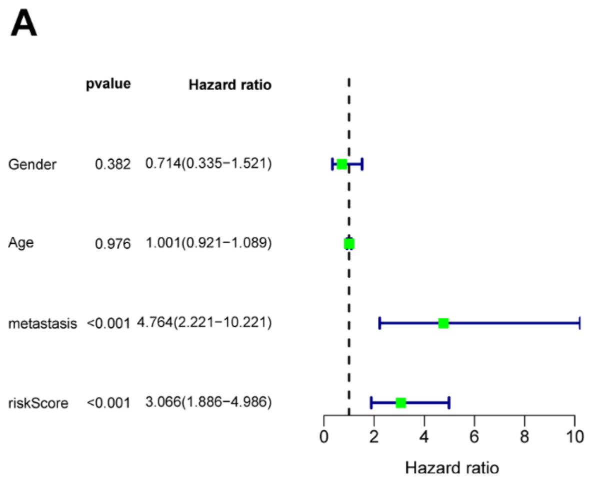

Supplementary Figure 12. Evaluation of clinical data and risk score for prognosis of osteosarcoma. (**A**) Results of single factor Cox analysis of TCGA osteosarcoma data. (**B**) Multivariate Cox analysis of TCGA osteosarcoma data.

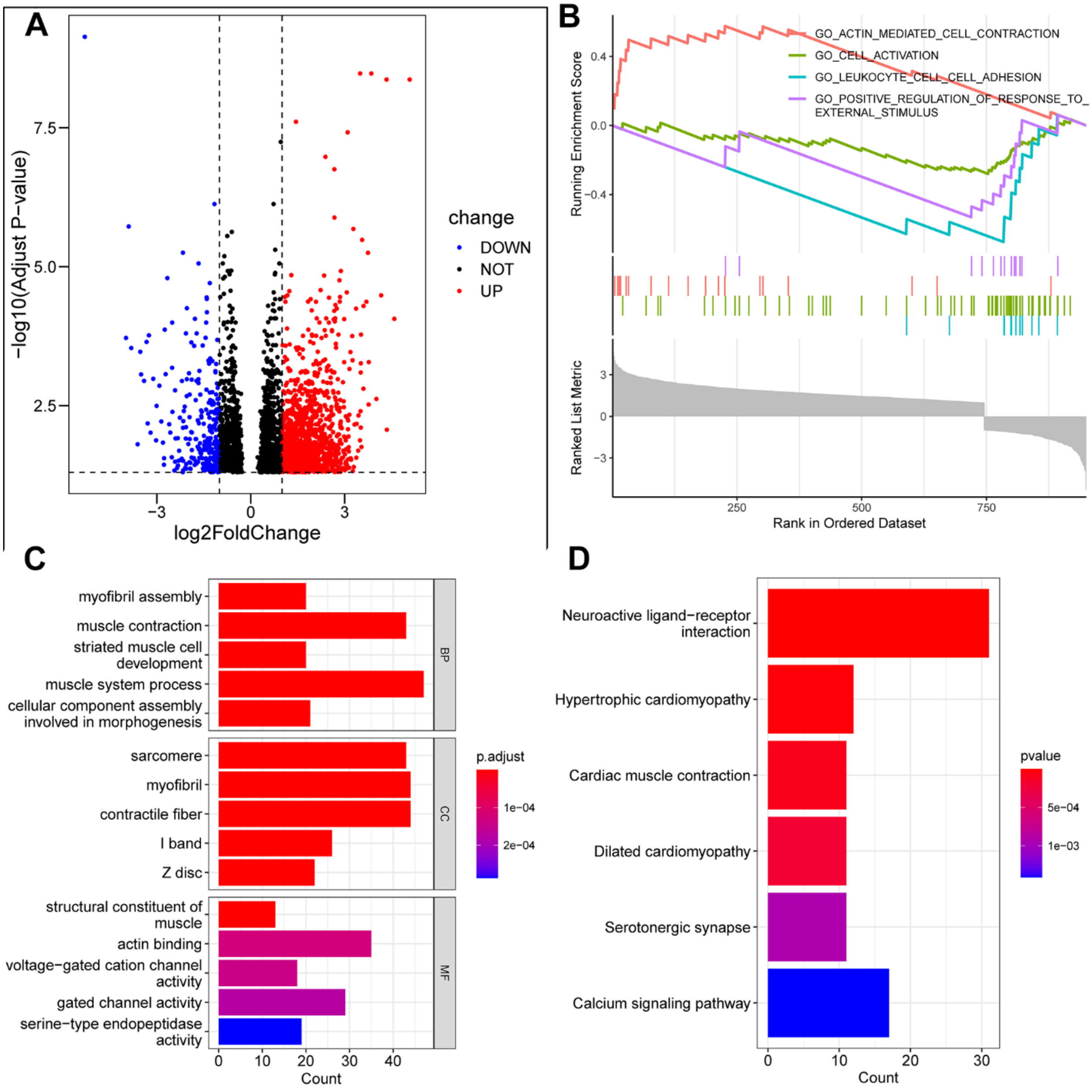

Supplementary Figure 13. Enrichment analysis. (**A**) Differential gene volcano map. (**B**) GSEA enrichment analysis. (**C**) GO enrichment analysis. (**D**) KEGG enrichment analysis.

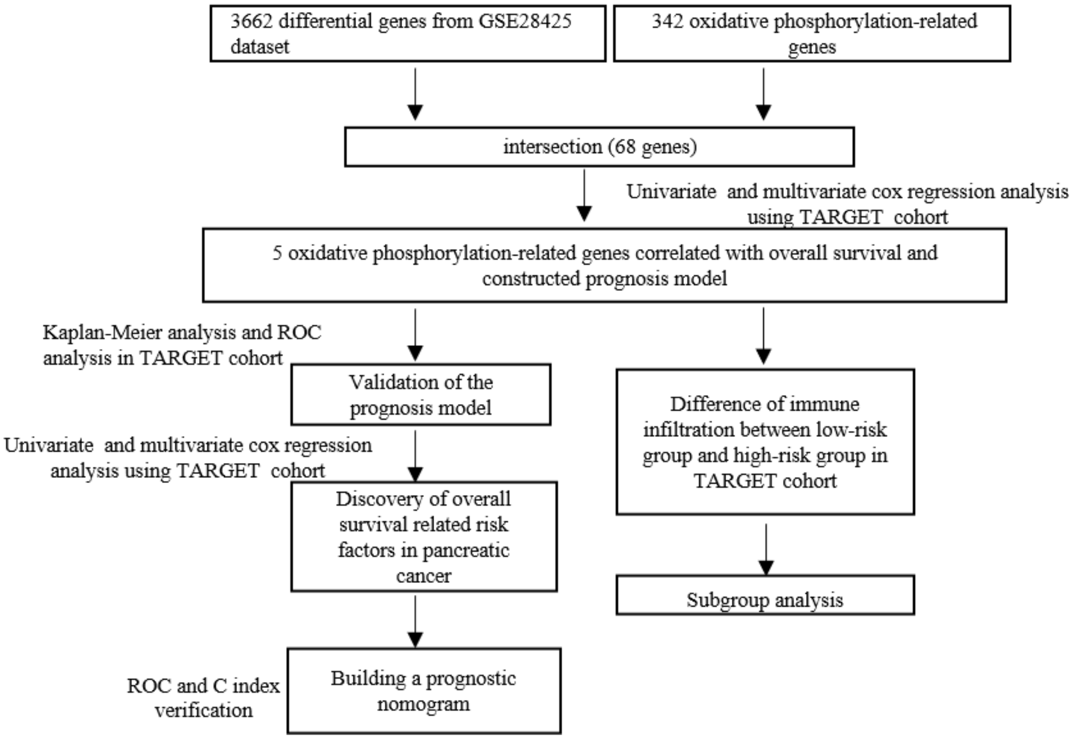

Supplementary Figure 14. Flow chart.
